# Predicting 90-Day Mortality in Locoregionally Advanced Head and Neck Squamous Cell Carcinoma after Curative Surgery

**DOI:** 10.3390/cancers10100392

**Published:** 2018-10-22

**Authors:** Lei Qin, Tsung-Ming Chen, Yi-Wei Kao, Kuan-Chou Lin, Kevin Sheng-Po Yuan, Alexander T. H. Wu, Ben-Chang Shia, Szu-Yuan Wu

**Affiliations:** 1 School of Statistics, University of International Business and Economics, Beijing 100029, China; qinlei@uibe.edu.cn; 2 Department of Otorhinolaryngology, Shuang-Ho Hospital, Taipei Medical University, New Taipei City 23561, Taiwan; 09326@s.tmu.edu.tw; 3 Graduate Institute of Business Administration, Fu Jen Catholic University, Taipei 116, Taiwan; kyw498762030@gmail.com; 4 Department of Oral and Maxillofacial Surgery, Wanfang Hospital, Taipei Medical University, Taipei 116, Taiwan; kclin0628@hotmail.com; 5 Department of Otorhinolaryngology, Wanfang Hospital, Taipei Medical University, Taipei 116, Taiwan; dryuank@gmail.com; 6 Ph.D. Program for Translational Medicine, Taipei Medical University, Taipei 116, Taiwan; chaw1211@tmu.edu.tw; 7 College of Management, Taipei Medical University, Taipei 106, Taiwan; 8 Department of Radiation Oncology, Wanfang Hospital, Taipei Medical University, Taipei 116, Taiwan; 9 Department of Internal Medicine, School of Medicine, College of Medicine, Taipei Medical University, Taipei 110, Taiwan

**Keywords:** comorbidity score, mortality, locoregionally advanced, HNSCC, curative surgery

## Abstract

*Purpose:* To propose a risk classification scheme for locoregionally advanced (Stages III and IV) head and neck squamous cell carcinoma (LA-HNSCC) by using the Wu comorbidity score (WCS) to quantify the risk of curative surgeries, including tumor resection and radical neck dissection. *Methods:* This study included 55,080 patients with LA-HNSCC receiving curative surgery between 2006 and 2015 who were identified from the Taiwan Cancer Registry database; the patients were classified into two groups, mortality (*n* = 1287, mortality rate = 2.34%) and survival (*n* = 53,793, survival rate = 97.66%), according to the event of mortality within 90 days of surgery. Significant risk factors for mortality were identified using a stepwise multivariate Cox proportional hazards model. The WCS was calculated using the relative risk of each risk factor. The accuracy of the WCS was assessed using mortality rates in different risk strata. *Results:* Fifteen comorbidities significantly increased mortality risk after curative surgery. The patients were divided into low-risk (WCS, 0–6; 90-day mortality rate, 0–1.57%), intermediate-risk (7–11; 2.71–9.99%), high-risk (12–16; 17.30–20.00%), and very-high-risk (17–18 and >18; 46.15–50.00%) strata. The 90-day survival rates were 98.97, 95.85, 81.20, and 53.13% in the low-, intermediate-, high-, and very-high-risk patients, respectively (log-rank *p* < 0.0001). The five-year overall survival rates after surgery were 70.86, 48.62, 22.99, and 18.75% in the low-, intermediate-, high-, and very-high-risk patients, respectively (log-rank *p* < 0.0001). *Conclusion:* The WCS is an accurate tool for assessing curative-surgery-related 90-day mortality risk and overall survival in patients with LA-HNSCC.

## 1. Introduction

The incidence of head and neck squamous cell carcinoma (HNSCC) in Taiwan is different from that in Western countries. Betel nut chewing is endemic to Taiwan and is observed in >90% patients with HNSCC in Taiwan [1,2,3,4,5]. Betel nut chewing results in a high risk of local recurrence and second primary HNSCC in patients with HNSCC in Taiwan [1,2,3,4,5]. Due to betel nut chewing, the proportion of oral cavity and nonoral cavity cancers in patients with HNSCC in Taiwan is approximately 66 and 34%, respectively [1,2,3,4,5,6]. The proportion of oral cavity cancers in patients with HNSCC is higher in Taiwan than in other countries [1,2,3,4,5,6]. Treatments for Taiwanese patients with HNSCC might be complicated, and the frequency of reirradiation is higher in Taiwan than in areas where betel nut chewing is not endemic [1,2,3,4,5]. Therefore, comprehensive curative surgery is the main treatment (accounting for 64.09% of all HNSCC treatments) for patients with HNSCC in Taiwan [1,2,3,4,5,6]. In addition, in Taiwan, at initial diagnosis, >50% of HNSCC cases are locoregionally advanced (Stages III and IV) HNSCC (LA-HNSCC) [6]. Instead of RT or chemotherapy, the initial treatment for LA-HNSCC is surgical resection of the primary tumor and neck dissection, followed by postoperative radiotherapy (RT) or concurrent chemoradiotherapy (CCRT).

Curative head and neck surgery, including radical neck dissection, is associated with a mortality rate of 1.5–8.5% [7,8,9,10,11]; however, the time interval between curative surgery and mortality has not been specified in the literature. Most studies on curative surgery for HNSCC were published in the 1970s to 1980s [7,8,9,10,11]; however, surgical techniques have improved considerably in the past 20 years [12,13]. Definitive data on mortality rates after curative surgery for LA-HNSCC in the past 20 years, particularly in Asia, are not available. In this study, we estimated mortality rates after curative surgery in patients with LA-HNSCC between 2006 and 2015. A new comorbidity score to predict mortality rates in patients with LA-HNSCC receiving curative surgery was also proposed because modern RT techniques, chemotherapy regimens, induction chemotherapy, and immune therapy might be more suitable alternative curative-intent treatments than curative surgery for high-mortality risk patients with LA-HNSCC [2,3,4,14,15,16,17,18,19,20,21].

The mean age of patients with HNSCC in Taiwan has been reported to be 55 years; consequently, the patients are generally individuals who provide the main economic support to their families [1,2,3,4,5,6]. We hope to reduce mortality rates after aggressive treatments in LA-HNSCC and propose a new comorbidity score to preoperatively predict 90-day mortality and overall survival in patients with LA-HNSCC who will receive curative surgery. The new comorbidity score can be used to determine whether curative surgery or other curative-intent aggressive treatments are the optimal treatment [2,4,14,15,16,17,18,19,20,21].

## 2. Patients and Methods

**Ethics approval and consent:** Our protocols were reviewed and approved by the Institutional Review Board of Taipei Medical University (TMU-JIRB No. 201712019).

### 2.1. Database

The study population was identified from the Taiwan Cancer Registry database (TCRD). The TCRD is a crucial research resource for epidemiological studies, and the results obtained using the database can be used as a reference when developing medical and health policies. The Cancer Registry database of Collaboration Center of Health Information Application contains detailed cancer-related information on clinical stages, RT doses, habits (smoking, betel nut chewing, and drinking), surgical procedures, techniques, and chemotherapy regimens [2,4,22,23]. The Institutional Review Board of Taipei Medical University approved this study (TMU-No. 201712019). The TCRD is released to the public for research purposes after identification numbers are scrambled and personal information is de-identified.

### 2.2. Selection of Patients and Controls

This study included 55,080 patients with LA-HNSCC who had received curative surgeries, including tumor resection and ipsilateral radical neck dissection, between 1 January 2006 and 31 December 2015. In the patients with HNSCC, clinical staging was performed according to the American Joint Committee on Cancer (AJCC), Seventh Edition. Squamous cell carcinoma was confirmed in all study patients identified from the TCRD. Patients with the following contraindications for curative surgery were excluded from the study: An Eastern Cooperative Oncology Group performance status of ≥2, a fixed neck mass in the deep cervical fascia, skull base involvement, circumferential or near circumferential involvement, and invasion of the carotid vessels if the patient could not tolerate a balloon occlusion test. All head and neck surgeons in Taiwan are head and neck oncology specialists certified by the Taiwan Ministry of Health and Welfare. We only included patients aged >18 years to restrict our study population to adults. Patients with metastatic HNSCC were excluded. The included patients were classified into two groups, namely mortality (*n* = 1287, mortality rate = 2.34%) and survival (*n* = 53,793, survival rate = 97.66%) groups, according to the event of mortality within 90 days after curative surgery. For each patient, the index date was designated as the date of curative surgery.

### 2.3. Statistical Analysis

All statistical analyses were performed using SAS statistical software (SAS for Windows, version 9.2, SAS Institute, Cary, NC, USA). Statistical significance was set at *p* ≤ 0.05.

For demographic characteristics, age group (18–29, 30–39, 40–49, 50–59, 60–69, and ≥70) and sex were selected as the basic information of the patients. Age was calculated as the time interval between the index date and birth date, and data on sex were extracted from the database. Comorbidities were evaluated using the Charlson comorbidity index (CCI), and before surgery, physical status was determined according to the American Society of Anesthesiologists (ASA) Physical Status Classification System [2,4,24,25,26]. Patients with recent (within 6 months before the index date) myocardial infarction (MI), cerebral vascular accident (CVA), transient ischemic attack (TIA), or coronal arterial disease (CADs) with stents, ongoing cardiac ischemia or severe valve dysfunction, severe reduction of ejection fraction, sepsis, disseminated intravascular coagulation (DIC), adult respiratory distress syndrome (ARDS), or end-stage renal disease (ESRD) were excluded from the study. Only comorbidities observed 6 months before the index date were included in the analysis; comorbidities were identified and included according to the main International Classification of Diseases, Ninth Revision, Clinical Modification (ICD-9-CM) diagnostic codes for the first admission or 3 or more repeated main diagnosis codes for visits to outpatient departments. The comorbidities of interest were diabetes mellitus (DM), hypertension (HTN), pneumonia, chronic obstructive pulmonary disease (COPD), hepatitis B (HBV) infection, hepatitis C (HCV) infection, implanted pacemaker, MI, CVA, TIA, CADs, angina, heart valve dysfunction, ESRD, sepsis, chronic kidney disease (CKD), heart failure, DIC, ARDS, aortic aneurysm, peripheral vascular disease (PVD), peptic ulcer disease (PUD), dementia, chronic pulmonary disease, connective tissue disease, mild liver disease, hemiplegia, moderate or severe renal disease, any non-HNSCC solid cancer, leukemia, lymphoma, moderate or severe liver disease, metastatic non-HNSCC solid cancer, previous thoracic surgery, smoking, obesity, asthma, and bowel obstruction. The chi-square test was used to compare demographic characteristics and comorbidities between the mortality and survival groups.

In this study, we aimed to identify significant risk factors for mortality within 90 days after curative surgery and proposed the Wu comorbidity score (WCS) to assess mortality risk associated with curative surgery in patients with HNSCC. Univariate and multivariate Cox proportional hazard models were constructed to calculate the hazard ratios (HRs) of the variables and corresponding 95% confidence intervals (CIs). A stepwise selection method was used to select all the variables that exerted significant effects on the survival duration in the patients. Variables with coefficients of >0 or HRs of >1 were selected as risk factors to construct the WCS by adding points according to the HRs. We divided all the patients into different strata according to the WCS and confirmed that the patients with high scores had high mortality risk after curative surgery. The cumulative mortality rate was estimated using the Kaplan–Meier method, and differences among the risk strata were determined using the log-rank test. Two-tailed *p* < 0.05 was considered statistically significant.

## 3. Results

Table 1 shows a comparison of demographic characteristics and mortality rates within 90 days after curative surgery between the mortality and survival groups. Significant differences were observed between the groups in the age (*p* < 0.0001), sex (*p* < 0.0001), and comorbidities, such as DM, HTN, and pneumonia.

Table 2 and Table 3 present the relative risk for each variable estimated using univariate and multivariate Cox proportional hazard models. Fewer variables were significant in the multivariate model than in the univariate model, indicating strong collinearity between the variables. Therefore, we used a stepwise method in the multivariate model for variable selection (Table 4). Among comorbidities, significant variables were HTN, pneumonia, COPD, sepsis, heart failure, DIC, ARDS, dementia, mild to severe liver disease, hemiplegia, moderate or severe renal disease, any tumor, and metastatic solid tumor.

**Figure 1:**
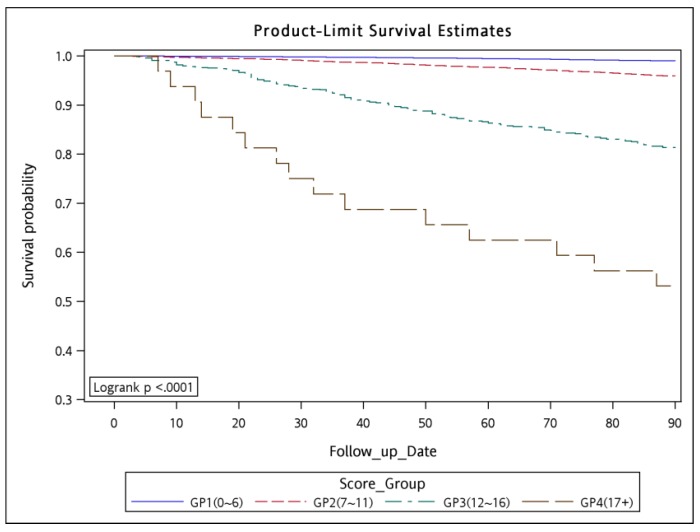
Kaplan–Meier curves for 90-day survival in patients with locoregionally advanced head and neck squamous cell carcinoma receiving curative surgery associated with the four risk groups. Note: *p*-value of Log Rank Test is <0.0001.

**Figure 2:**
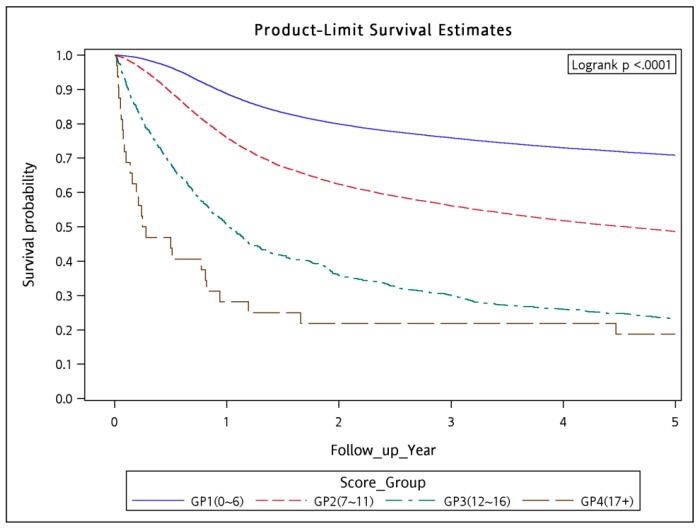
Kaplan–Meier curves for five years overall survival in patients with locoregionally advanced head and neck squamous cell carcinoma receiving curative surgery associated with the four risk groups. Note: *p*-value of Log Rank Test is <0.0001.

The WCS was calculated using significant variables other than HTN because the HR of HTN was <1. Although HTN increased the risk of outcomes, which can be observed from univariate analysis (Table 2, HR = 1.356, CI: 1.211–1.518), collinearity in multivariate model may have reduced the HR to <1 (Table 4, HR = 0.803, CI: 0.708–0.91), which is a common statistical phenomenon. We calculated the WCS by adding points according to the HR of each risk factor. The points of each risk factor were assigned as the largest integer less than or equal to its HR (last column in Table 4); for example, 2 points for pneumonia with an HR of 2.092 and 3 points for ARDS with an HR of 3.897. In the WSC, a high number of points were assigned to risk factors with high relative mortality risk within 90 days. In our study, the minimum and maximum values of the WCS were 0 and 18+, respectively. We collapsed the range of the WCS into 4 strata, namely the low-risk (WCS, 0–6; 90-day mortality rate, 0–1.57%), intermediate-risk (7–11; 2.71–9.99%), high-risk (12–16, 17.30–20.00%), and very-high-risk (17 to 18+; 46.15–50.00%) strata (Table 4). We used the CCI for scoring to predict 90-day mortality compared with the current scoring system (Figures S1 and S2), the risk groups of CCI were not feasible for predicting 90-day mortality in LA-HNSCC patients receiving curative surgery and could not reach statistical significance. In addition, there were scarcely LA-HNSCC patients with ASA classifications I and IV–V receiving curative surgery in our database. Therefore, we cannot use ASA classifications I–V to predict 90-day mortality in LA-HNSCC patients receiving curative surgery. The 90-day mortality rate tended to increase as the WCS increased, indicating the accuracy of the WCS. The 90-day mortality rate and five-year survival in the patients were estimated using the Kaplan–Meier method to analyze the risk of mortality associated with the 4 risk strata (Figure 1 and Figure 2). The 90-day survival rates were 98.97, 95.85, 81.20, and 53.13% in the low-, intermediate-, high-, and very-high-risk strata, respectively (log-rank test *p* < 0.0001; Figure 1). The five-year overall survival rates were 70.86, 48.62, 22.99, and 18.75% in the low-, intermediate-, high-, and very-high-risk strata, respectively (log-rank *p* < 0.0001; Figure 2).

## 4. Discussion

According to the Taiwan Cancer Registry report, 2017 edition [6], >90% of curative surgery procedures for LA-HNSCC are conducted in top-ranking medical centers. The ranking is based on accreditation of hospitals in Taiwan into 4 levels since 1988 (medical center, regional hospitals, local hospital, clinics); the accreditation grade affects the service quality and specific patient volume of the hospital [6]. Most curative surgery procedures were performed in hospitals with sufficient patient volume (>100 newly diagnosed patients with LA-HNSCC per year), thus leading to consistent patient outcomes for LA-HNSCC in Taiwan [6,27,28,29,30]. Therefore, in Taiwan, the overall mortality rate within 90 days after curative surgery in the patients with LA-HNSCC was only 2.34% (Table 5) after consultation with a professional head and neck surgeon and anesthesia consultation. In Table 1, <3% of the patients with LA-HNSCC and heart valve dysfunctions and <5% of the patients with LA-HNSCC and moderate or severe liver disease received curative surgery. Surgeons were unwilling to perform curative surgery on the patients with LA-HNSCC and ESRD. However, the 90-day mortality remained 2.34%. Therefore, we wanted to develop a highly accurate predictor score to estimate mortality rates after curative surgery because CCRT or induction chemotherapy, followed by CCRT, might be an alternative treatment for patients with LA-HNSCC [3,4]. The proportion of the patients with LA-HNSCC with smoking habit (approximately 90%) in Table 1 was consistent with that reported in previous studies in Taiwan [1,2,3,4,5]. The 90-day mortality rate was proportional to age, particularly in the patients aged >70 years. This is the first study to show that age is a predictor of 90-day mortality in the patients with LA-HNSCC after curative surgery (Table 1, Table 2 and Table 3).

Univariate and multivariate analyses revealed that age is an independent predictor of 90-day mortality after curative surgery in patients (Table 2 and Table 3). The male patients had higher 90-day mortality risk than did the female patients after curative surgery. These findings are consistent with those of previous studies, which reported endpoints different from those in our study [31,32]. From Table 3, the patients with LA-HNSCC and pneumonia or COPD exhibited high mortality rates. These findings are consistent with those of previous studies [33,34]. However, this is the first study to demonstrate that preoperative pneumonia increases mortality rates in the patients with LA-HNSCC who received curative surgery. Notably, although heart valve dysfunction, MI, CVA, TIA, angina, or CADs were listed as risk factors in the ASA Physical Status Classification System before surgery [24,26], these factors were not risk factors for 90-day mortality in our study. This discrepancy can be explained by our inclusion of comorbidities observed >6 months before the index date and exclusion of the comorbidities observed within 6 months of the index date. The mortality rates of these acute vascular diseases might decrease considerably after 6 months of having these diseases [35,36,37,38]. This is the first study to show the absence of correlations between heart valve dysfunction, MI, CVA, TIA, angina, or CADs and 90-day mortality rates associated with curative surgery in the patients with LA-HNSCC. These findings are reliable references for head and neck surgeons in the future.

Notably, HF, DIC, and ARDS were independent risk factors for 90-day mortality, even when comorbidities observed 6 months before the index date were included. This is because HF, DIC, and ARDS are chronic diseases [39,40,41,42] and not acute vascular diseases. In addition, HF, DIC, and ARDS were also listed in the ASA Physical Status Classification System before surgery [24,26]. Dementia and hemiplegia might affect self-care by patients who receive surgery and might result in an increased mortality rate after surgery [31,43]. Our study is the first to demonstrate that dementia and hemiplegia were independent risk factors for mortality in the patients with LA-HNSCC who had received head and neck curative surgery. Head and neck surgeons should carefully consider curative surgery for patients with LA-HNSCC and dementia or hemiplegia. Furthermore, in our study, liver disease or renal disease were independent risk factors for 90-day mortality. Moreover, Cramer et al. showed that liver disease increases the risk of perioperative mortality in patients with HNSCC, and this risk should be carefully considered during surgical decision-making and postoperative care [44]. ESRD was also an independent 90-day mortality risk factor in our study; this finding is consistent with the results of a previous study, which reported a slightly different endpoint from ours [45]. However, leukemia and lymphoma were not risk factors for 90-day mortality in our study (Table 3). Most patients with leukemia or lymphoma have long survival durations of >1 year [46,47]; this long survival duration might explain why leukemia and lymphoma did not affect 90-day mortality in our study. By contrast, non-HNSCC cancer with or without metastasis was an independent risk factor for 90-day mortality (Table 3). This result may be attributable to the weakening of overall physical health, immunity, and the hematological system owing to previous cancer treatments, such as systemic chemotherapy, major surgical procedures, or RT, which increase 90-day mortality rates because of systemic infection complications, hospitalizations, and uncontrolled coagulation or hematological problems [48,49,50]. For patients with LA-HNSCC and non-HNSCC cancer with or without metastasis, alternative curative-intent aggressive treatments might be considered [4].

In our analysis, the WCS corresponded with not only the 90-day mortality rates but also with the overall survival rates (Figure 2). Developing a new comorbidity score for predicting 90-day mortality in patients with LA-HNSCC who will receive curative surgery is currently valuable because of the evolution of contemporary chemotherapy, RT techniques, target therapy, or immunotherapy, particularly in the past 10 years [14,15,16,17,18,19,20,21]. An increasing number of alternative curative-intent aggressive treatments are available for patients with LA-HNSCC [14,15,16,17,18,19,20,21].

Because the development of new surgical procedures has minimized surgical morbidity and mortality, the contraindications to curative surgery for LA-HNSCC remain controversial [12,13]. However, patients with LA-HNSCC who have a high surgical risk because of comorbidities and whose condition cannot be optimized preoperatively should not be considered for new surgical procedures. Even after treatment by a professional head and neck surgeon and careful anesthesia consultation, the 90-day mortality in Taiwan remained at 2.34% from 2006 to 2015 (Table 5). The WCS can serve as a valuable tool for preoperative prediction of the risk associated with curative surgery in patients with LA-HNSCC. After predicting the risk, other alternative curative-intent aggressive treatments can be considered in LA-HNSCC patients [4,14,15,16,17,18,19,20,21].

To the best of our knowledge, our study is the first to use a comorbidity score to predict the 90-day mortality in the patients with LA-HNSCC who had received curative surgery. Figures revealed significant differences between low-, intermediate-, high-, and very-high-risk strata. These findings suggest that the WCS is a valid and specific tool for predicting 90-day and overall mortality in patients with LA-HNSCC who will receive curative surgery. Our literature review also revealed that our study also had the largest sample size among the studies that have proposed new comorbidity scores in the past 10 years.

This study has some limitations. First, the morbidity of curative surgery could not be determined because of differences in the levels of experience among surgeons and across hospitals; therefore, head and neck curative-surgery-related mortality estimates may have been biased. However, the Taiwan Cancer Registry report, 2017 edition, revealed that curative surgery for LA-HNSCC in Taiwan are mostly performed in hospitals with high patient volumes and large medical centers [6]. Therefore, the outcomes of head and neck curative surgery would be consistent in Taiwan. Second, because all the patients with LA-HNSCC were enrolled from an Asian population and all the surgical procedures were performed by Taiwanese surgeons, the corresponding ethnic and regional susceptibility to this disease remain unclear; hence, our results should be cautiously extrapolated to non-Asian populations. Third, the diagnoses of all comorbid conditions were based on ICD-9-CM codes. However, the Taiwan Cancer Registry administration randomly reviews charts and interviews patients to verify the accuracy of the diagnoses. Hospitals with outlier chargers or practices may be audited and be subsequently heavily penalized if malpractice or discrepancies are identified. In addition, the quality and precision of ICD-9-CM codes in Taiwan have been verified and proven by previous studies [51,52]. Therefore, to obtain accurate information on population specificity and disease occurrence, large-scale randomized trials that compare carefully selected patients who had received suitable treatments are required. Fourth, we have scarcely very-high WCS patients (32/55,080 = 0.05%) and scarcely high WCS patients (<1000/55,080 = 1.60%). For remaining more than 98 percent of the patients only two risk strata groups are left and this is the same problem what we have with the ASA II and ASA III patients. Nevertheless, the individual 90-day mortality can be predicted upon the findings of this study because a big cancer registry supports this data. Finally, the TCRD does not contain information on dietary habits, socioeconomic status, or body mass index, which may all be risk factors for mortality. However, considering the magnitude and statistical significance of the effects observed in this study, these limitations are unlikely to have affected the conclusions.

## 5. Conclusions

The WCS is a valid tool for predicting 90-day mortality and overall survival in patients with LA-HNSCC who will receive curative surgery. Other alternative curative-intent aggressive treatments can be considered for patients with LA-HNSCC in the high- to very-high-risk strata instead of curative surgery.

## Figures and Tables

**Table 1:** Demographic characteristics between death within 90 days and survival groups receiving curative surgery in locoregionally advanced head and neck squamous cell carcinoma patients.

Factor	Death No.	Death Rate	Survival No.	Survival Rate	*p* Value
Number of patients	1287		53,793		
Age (years)					<0.0001
18–29	2	0.16%	696	1.29%	
30–39	40	3.11%	4978	9.25%	
40–49	231	17.95%	14,379	26.73%	
50–59	352	27.35%	16,611	30.88%	
60–69	263	20.44%	10,046	18.68%	
≥70	399	31.00%	7083	13.17%	
Sex					<0.0001
Female	195	7.38%	5697	10.59%	
Male	1192	92.62%	48,096	89.41%	
Comorbidity					
DM	80	6.22%	1804	3.35%	<0.0001
HTN	477	37.06%	16,260	30.23%	<0.0001
Pneumonia	250	19.43%	2665	4.95%	<0.0001
COPD	240	18.65%	4517	8.40%	<0.0001
Hepatitis B	8	0.62%	425	0.79%	0.4989
Hepatitis C	32	2.49%	1003	1.86%	0.1045
Implanted pacemaker	2	0.16%	22	0.04%	0.0518
MI, CVA, TIA, angina, or CAD	280	21.76%	6957	12.93%	<0.0001
Heart valve dysfunction	35	2.72%	659	1.23%	<0.0001
ESRD	0	0.00%	0	0.00%	
Sepsis	172	13.36%	882	1.64%	<0.0001
CKD	165	12.82%	1754	3.26%	<0.0001
Heart failure	92	7.15%	1000	1.86%	<0.0001
DIC	5	0.39%	5	0.01%	<0.0001
ARDS	4	0.31%	18	0.03%	<0.0001
Aortic aneurysm	4	0.31%	58	0.11%	0.0319
PVD	33	2.56%	707	1.31%	0.0001
PUD	268	20.82%	7394	13.75%	<0.0001
Dementia	71	5.52%	1100	2.04%	<0.0001
Chronic pulmonary disease	222	17.25%	4810	8.94%	<0.0001
Connective tissue disease	23	1.79%	614	1.14%	0.0323
Mild liver disease	255	19.81%	7733	14.38%	<0.0001
Hemiplegia	107	8.31%	1970	3.66%	<0.0001
Moderate or severe renal disease	166	12.90%	1757	3.27%	<0.0001
Any non-HNSCC Solid Cancer	659	51.20%	14,591	27.12%	<0.0001
Leukemia	2	0.16%	33	0.06%	0.1858
Lymphoma	22	1.71%	647	1.20%	0.101
Moderate or severe liver disease	89	6.92%	2487	4.62%	0.0001
Metastatic non-HNSCC solid cancer	548	42.58%	13,390	24.89%	<0.0001
Smoking	1158	89.98%	48,413	90.00%	0.8989
Previous thoracic surgery	6	0.47%	268	0.50%	0.6767
Obesity	11	1.24%	699	1.30%	0.7756
Asthma	10	0.78%	429	0.80%	0.9573
Bowel obstruction	3	0.23%	123	0.23%	0.8457

Diabetes mellitus: DM; hypertension: HTN; Chronic Obstructive Pulmonary Disease: COPD; Hepatitis B: HBV; Hepatitis C: HCV; myocardial infarction: MI; cerebral vascular accident: CVA; transient ischemic attack: TIA; coronal arterial disease: CAD; end stage renal disease: ESRD; Chronic kidney disease: CKD; disseminated intravascular coagulation: DIC; adult respiratory distress syndrome: ARDS; peripheral vascular disease: PVD; peptic ulcer disease: PUD.

**Table 2:** Mortality risk assessment through univariate Cox proportional hazard model in locoregionally advanced head and neck squamous cell carcinoma patients receiving curative surgery.

Factor	HR	95% CI	*p* Value
Age (years)			
18–29	1	(Reference)	
≥30	1.277	2.077, 32.989	0.0027
≥40	1.463	2.546, 4.709	<0.0001
≥50	2.19	1.916, 2.503	<0.0001
≥60	2.245	2.012, 2.504	<0.0001
≥70	2.915	2.59, 3.28	<0.0001
Sex			
Female	1	(Reference)	
Male	1.48	1.201, 1.824	0.0002
Comorbidities			
DM	1.894	1.51, 2.374	<0.0001
HTN	1.356	1.211, 1.518	<0.0001
Pneumonia	4.476	3.898, 5.138	<0.0001
COPD	2.466	2.143, 2.838	<0.0001
Hepatitis B	0.787	0.393, 1.576	0.4986
Hepatitis C	1.342	0.945, 1.905	0.1004
Implanted pacemaker	3.716	0.928, 14.874	0.0636
MI, CVA, TIA, angina, or CAD	1.857	1.627, 2.12	<0.0001
Heart valve dysfunction	2.224	1.589, 3.111	<0.0001
Sepsis	8.647	7.364, 10.153	<0.0001
CKD	4.245	3.605, 4.999	<0.0001
Heart failure	3.965	3.207, 4.902	<0.0001
DIC	32.834	13.643, 79.019	<0.0001
ARDS	9.058	3.395, 24.166	<0.0001
Aortic aneurysm	2.818	1.056, 7.519	0.0385
PAD	1.962	1.389, 2.772	0.0001
PUD	1.64	1.433, 1.876	<0.0001
Dementia	2.735	2.153, 3.475	<0.0001
Chronic pulmonary disease	2.1	1.817, 2.427	<0.0001
Connective tissue disease	1.567	1.037, 2.367	0.0328
Mild liver disease	1.465	1.277, 1.68	<0.0001
Hemiplegia	2.35	1.928, 2.865	<0.0001
Moderate or severe renal disease	4.272	3.629, 5.028	<0.0001
Any non-HNSCC Solid Cancer	2.753	1.917, 3.953	<0.0001
Leukemia	2.506	0.626, 10.032	0.1941
Lymphoma	1.423	0.934, 2.168	0.1008
Moderate or severe liver disease	1.525	1.229, 1.891	0.0001
Metastatic non-HNSCC solid cancer	2.21	1.979, 2.468	<0.0001
Smoking	0.964	0.405, 1.459	0.3571
Previous thoracic surgery	1.168	0.778, 2.589	0.6734
Obesity	1.473	0.746, 1.896	0.8197
Asthma	1.384	0.804, 8.09	0.7592
Bowel obstruction	1.132	0.494, 2.873	0.8112

Diabetes mellitus: DM; hypertension: HTN; Chronic Obstructive Pulmonary Disease: COPD; Hepatitis B: HBV; Hepatitis C: HCV; myocardial infarction: MI; cerebral vascular accident: CVA; transient ischemic attack: TIA; coronal arterial disease: CAD; end stage renal disease: ESRD; Chronic kidney disease: CKD; disseminated intravascular coagulation: DIC; adult respiratory distress syndrome: ARDS; peripheral vascular disease: PVD; peptic ulcer disease: PUD.

**Table 3:** Mortality risk assessment through multivariate Cox proportional hazard model in locoregionally advanced head and neck squamous cell carcinoma patients receiving curative surgery.

Factor	HR	95% CI	*p* Value
Age (years)			
18–29	1	(Reference)	
≥30	1.012	0.486, 3.336	0.3348
≥40	1.125	1.304, 2.555	0.0004
≥50	1.309	1.107, 1.547	0.0016
≥60	1.218	1.037, 1.432	0.0166
≥70	1.902	1.618, 2.236	<0.0001
Sex			
Female	1	(Reference)	
Male	1.439	1.163, 1.78	0.0008
Comorbidities			
DM	1.188	0.942, 1.5	0.1463
HTN	0.811	0.713, 0.922	0.0014
Pneumonia	2.093	1.79, 2.447	<0.0001
COPD	1.262	1.007, 1.581	0.0431
Hepatitis B	0.864	0.429, 1.741	0.6823
Hepatitis C	0.746	0.488, 1.142	0.1772
Implanted pacemaker	1.453	0.36, 5.873	0.6001
MI, CVA, TIA, angina, or CAD	0.891	0.745, 1.066	0.2072
Heart valve dysfunction	1.215	0.858, 1.721	0.2716
Sepsis	4.079	3.418, 4.869	<0.0001
CKD	1.117	0.658, 1.897	0.6818
Heart failure	2.037	1.617, 2.567	<0.0001
DIC	7.585	3.105, 18.53	<0.0001
ARDS	4.04	1.494, 10.923	0.0059
Aortic aneurysm	1.059	0.394, 2.845	0.9093
PAD	1.107	0.777, 1.578	0.5733
PUD	1.063	0.923, 1.225	0.3953
Dementia	1.583	1.234, 2.029	0.0003
Chronic pulmonary disease	0.97	0.774, 1.216	0.7916
Connective tissue disease	1.173	0.772, 1.781	0.4551
Mild liver disease	1.211	1.043, 1.407	0.0121
Hemiplegia	1.426	1.117, 1.821	0.0044
Moderate or severe renal disease	2.092	1.235, 3.544	0.0061
Any non-HNSCC solid cancer	2.306	1.599, 3.325	<0.0001
Leukemia	1.989	0.496, 7.981	0.332
Lymphoma	1.455	0.952, 2.225	0.0832
Moderate or severe liver disease	1.212	1.104, 1.519	0.0025
Metastatic non-HNSCC solid Cancer	2.144	1.916, 2.399	<0.0001
Smoking	0.846	0.618, 1.126	0.5753
Previous thoracic surgery	0.957	0.901, 1.976	0.8251
Obesity	1.015	0.879, 1.705	0.9088
Asthma	1.203	0.798, 1.511	0.8603
Bowel obstruction	1.047	0.505, 1.984	0.9221

Diabetes mellitus: DM; hypertension: HTN; Chronic Obstructive Pulmonary Disease: COPD; Hepatitis B: HBV; Hepatitis C: HCV; myocardial infarction: MI; cerebral vascular accident: CVA; transient ischemic attack: TIA; coronal arterial disease: CAD; end stage renal disease: ESRD; Chronic kidney disease: CKD; disseminated intravascular coagulation: DIC; adult respiratory distress syndrome: ARDS; peripheral vascular disease: PVD; peptic ulcer disease: PUD.

**Table 4:** Stepwise selection results and comorbidity score for multivariate Cox proportional hazard model in locoregionally advanced head and neck squamous cell carcinoma patients receiving curative surgery.

Factor	HR	95% CI	*p* Value	Points
Age (years)				
≥40	1.913	1.376, 2.659	0.0001	1
≥50	1.309	1.107, 1.547	0.0016	1
≥60	1.221	1.039, 1.435	0.0154	1
≥70	1.894	1.613, 2.225	<0.0001	1
Sex				
Female	1	(Reference)		
Male	1.425	1.153, 1.76	0.001	1
Comorbidities				
HTN	0.803	0.708, 1.11	0.1006	0
Pneumonia	2.092	1.79, 2.446	<0.0001	2
COPD	1.227	1.052, 1.431	0.0093	1
Sepsis	4.161	3.492, 4.958	<0.0001	4
Heart failure	2.056	1.646, 2.566	<0.0001	2
DIC	7.683	3.152, 18.728	<0.0001	7
ARDS	3.897	1.444, 10.52	0.0073	3
Dementia	1.598	1.247, 2.048	0.0002	1
Mild liver disease	1.251	1.087, 1.439	0.0018	1
Hemiplegia	1.342	1.089, 1.654	0.0059	1
Moderate or severe renal disease	2.361	1.983, 2.81	<0.0001	2
Any non-HNSCC solid cancer	2.289	1.588, 3.3	<0.0001	2
Moderate or severe liver disease	1.284	1.034, 1.473	0.0083	1
Metastatic non-HNSCC solid cancer	2.142	1.915, 2.397	<0.0001	2

Diabetes mellitus: DM; hypertension: HTN; Chronic Obstructive Pulmonary Disease: COPD; Hepatitis B: HBV; Hepatitis C: HCV; myocardial infarction: MI; cerebral vascular accident: CVA; transient ischemic attack: TIA; coronal arterial disease: CAD; end stage renal disease: ESRD; Chronic kidney disease: CKD; disseminated intravascular coagulation: DIC; adult respiratory distress syndrome: ARDS; peripheral vascular disease: PVD; peptic ulcer disease: PUD.

**Table 5:** Mortality (%) by different cumulative comorbidity scores among locoregionally advanced head and neck squamous cell carcinoma patients receiving curative surgery.

Score	No of Patient	No of Death	Death Rate
0	107	0	0.00%
1	277	0	0.00%
2	1104	5	0.45%
3	4289	16	0.37%
4	8948	67	0.75%
5	11,317	124	1.10%
6	10,621	167	1.57%
7	8423	228	2.71%
8	4621	168	3.64%
9	2514	154	6.13%
10	1263	109	8.63%
11	681	68	9.99%
12	393	68	17.30%
13	236	44	18.64%
14	149	32	21.48%
15	70	15	21.43%
16	35	7	20.00%
17	26	12	46.15%
18+	6	3	50.00%
Total	55,080	1287	2.34%

## Supplementary Materials

The following are available online at http://www.mdpi.com/2072-6694/10/10/392/s1. Figure S1: Kaplan–Meier curves for 90-day survival in patients with locoregionally advanced head and neck squamous cell carcinoma receiving curative surgery associated with the four risk groups from Charlson Comorbidity Index, Figure S2: Kaplan–Meier curves for 90-day survival in patients with locoregionally advanced head and neck squamous cell carcinoma receiving curative surgery associated with the four risk groups from Wu comorbidity score.

## Abbreviations

AJCC	American Joint Committee on Cancer
ARDS	Adult Respiratory Distress Syndrome
ASA	American Society of Anesthesiologists
CAD	Coronal Arterial Disease
CCI	Charlson Comorbidity Index
CCRT	Concurrent Chemoradiotherapy
CI	Confidence Interval
CKD	Chronic Kidney Disease
COPD	Chronic Obstructive Pulmonary Disease
CVA	Cerebral Vascular Accident
DIC	Disseminated Intravascular Coagulation
ESRD	End-Stage Renal Disease
HBV	Hepatitis B
HCV	Hepatitis C
HNSCC	Head and Neck Squamous Cell Carcinoma
HR	Hazard Ratio
HTN	Diabetes Mellitus DM Hypertension
ICD-9-CM	International Classification of Diseases Ninth Revision Clinical Modification
LA-HNSCC	Locoregionally Advanced Head and Neck Squamous Cell Carcinoma
MI	Myocardial Infarction
PUD	Peptic Ulcer Disease
PVD	Peripheral Vascular Disease
RT	Radiotherapy
TCRD	Taiwan Cancer Registry Database
TIA	Transient Ischemic Attack
