# Supplementary material for: Predicting 90-Day Mortality in Locoregionally Advanced Head and Neck Squamous Cell Carcinoma after Curative Surgery
Source: Cancers (Basel). 2018 Oct 22;10(10):392. doi: 10.3390/cancers10100392 (PMC6210656; doi:10.3390/cancers10100392)
Supplement: Supplementary file 1 [file cancers-10-00392-s001.pdf]

Supplementary Materials: Predicting 90-Day Mortality in Locoregionally Advanced Head and Neck Squamous Cell Carcinoma after Curative Surgery

Lei Qin; Yi-Wei Kao; Tsung-Ming Chen; Kuan-Chou Lin; Kevin Sheng-Po Yuan; Alexander T.H. Wu; Ben-Chang Shia and Szu-Yuan Wu

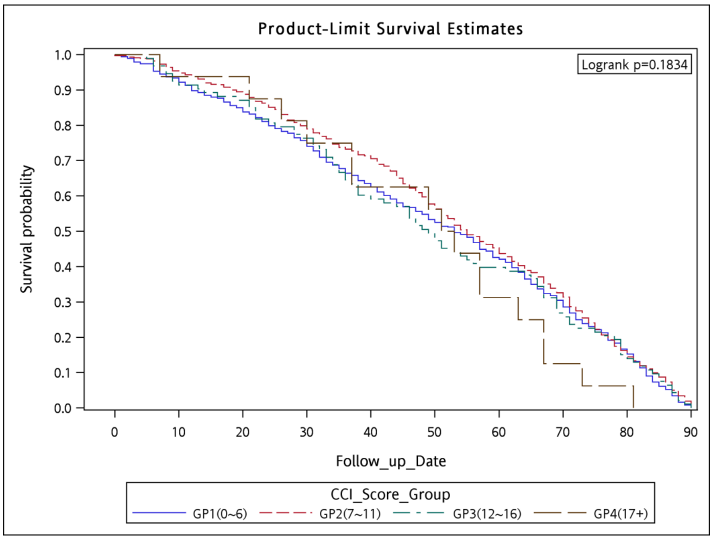

Figure S1. Kaplan–Meier curves for 90-day survival in patients with locoregionally advanced head and neck squamous cell carcinoma receiving curative-surgery associated with the four risk groups from Charlson Comorbidity Index. Note: *p*-value of Log Rank Test is <0.1834.

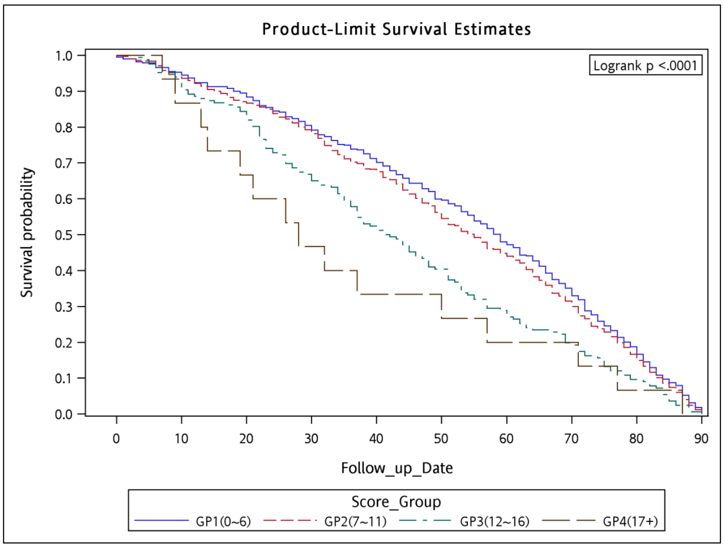

Figure S2. Kaplan–Meier curves for 90-day survival in patients with locoregionally advanced head and neck squamous cell carcinoma receiving curative-surgery associated with the four risk groups from Wu comorbidity score Note: *p*-value of Log Rank Test is <0.0001.

© 2018 by the authors. Licensee MDPI, Basel, Switzerland. This article is an open access article distributed under the terms and conditions of the Creative Commons Attribution (CC BY) license (http://creativecommons.org/licenses/by/4.0/).
